# Integrative *in silico* and *in vitro* analysis of *Pinus roxburghii* essential oil: unveiling its antioxidant, antidiabetic, and antiglycation potential

**DOI:** 10.3389/fphar.2025.1554562

**Published:** 2025-04-29

**Authors:** Rubina Naz Qaisrani, Shah Iram Niaz, Raheela Bano, Hatem A. Abuelizz, Arooj Mohsin Alvi, Sadia Chaman, Qaiser Aziz, Muhammad Asif Wazir, Muhammad Akram, Saiqa Ishtiaq, Muhammad Ramzan, Adnan Amin

**Affiliations:** ^1^ Institute of Chemical Sciences, Gomal University, Dera Ismail Khan, Pakistan; ^2^ Natural Products Research Lab (NPRL), Department of Pharmacognosy, Faculty of Pharmacy, Gomal University, Dera Ismail Khan, Pakistan; ^3^ Department of Pathology, Gomal Medical College, Dera Ismail Khan, Pakistan; ^4^ Department of Pharmaceutical Chemistry, College of Pharmacy, King Saud University, Riyadh, Saudi Arabia; ^5^ Faculty of Pharmacy, IBADAT International University, Islamabad, Pakistan; ^6^ Institute of Pharmaceutical Sciences, University of Veterinary & Animal Sciences (UVAS), Lahore, Pakistan; ^7^ Faculty of Health and Medical Sciences, University of Azad Jammu and Kashmir Muzaffarabad, Pakistan; ^8^ Faculty of Pharmacy, Department of Pharmacognosy, The Islamiya University of Bahawalpur, Punjab, Pakistan; ^9^ Pakistan Council for Scientific and Industrial Research (PCSIR), Bahawalpur, Peshawar, Pakistan; ^10^ Centre for Study of Human Health, Emory University, Atlanta, GA, United States; ^11^ Faculty of Pharmacy and Allied Health Sciences, Iqra University, Islamabad, Pakistan; ^12^ Department of Life Sciences, Yeungman University, Gyeongsan, South Korea

**Keywords:** oxidative stress, monoterpenes, α-glucosidase, AGEs, β-amyloid

## Abstract

**Introduction:**

Essential Oils (EOs) are a rich source of secondary metabolites that exhibit various biological activities.

**Methods:**

This study includes GCMS analysis of *Pinus roxburghii* EO, computational investigation including drug likeness, ADMET properties, molecular docking, and *in vitro* evaluations for possible antioxidant, antidiabetic, and anti‐AGEs actions.

**Results:**

GCMS analysis identified β‐pinene (30%) as major component, succeeded by caryophyllene (29.37%), o‐xylene (8.98%), aromadendrine (8.29%), and α‐himachalene (6.82%). Molecular docking showed significant interaction of transcription regulators 1JIJ with Caryophyllene oxide (ΔG ‐7.5 (kJ mol‐1), 3TOP with α‐himachalene (ΔG ‐6.8 (kJ mol‐1) and 4F5S with α‐himachalene (ΔG ‐7.7 (kJ mol‐1). The EO exhibited elevated phenolic content (26.3±0.45 mg/G GAE) and considerable antioxidant capacity in DPPH (14.2±0.62), H2O2 (73.3±1.7), and FRAP assays (312±14.6 μg). The antidiabetic assays demonstrated a notable inhibition of α‐glucosidase (IC50 0.12 mg/mL) and advanced glycation end products (AGEs) in both non‐oxidative (IC50 0.052 mg/mL) and oxidative modes (IC50 1.61 mg/mL). During mechanistic investigations it was observed that EO exerts a protective effect against β‐amyloid formation and significantly entraps carbonyl moieties.

**Conclusions:**

It was observed that P. roxburghii EO has notable antidiabetic and anti-AGEs properties and these finding support a good potential for management of diabetes and allied co-morbidities. In future formulation design studies can be helpful for pharmaceutical industry to opt the formulation.

## Graphical Abstract

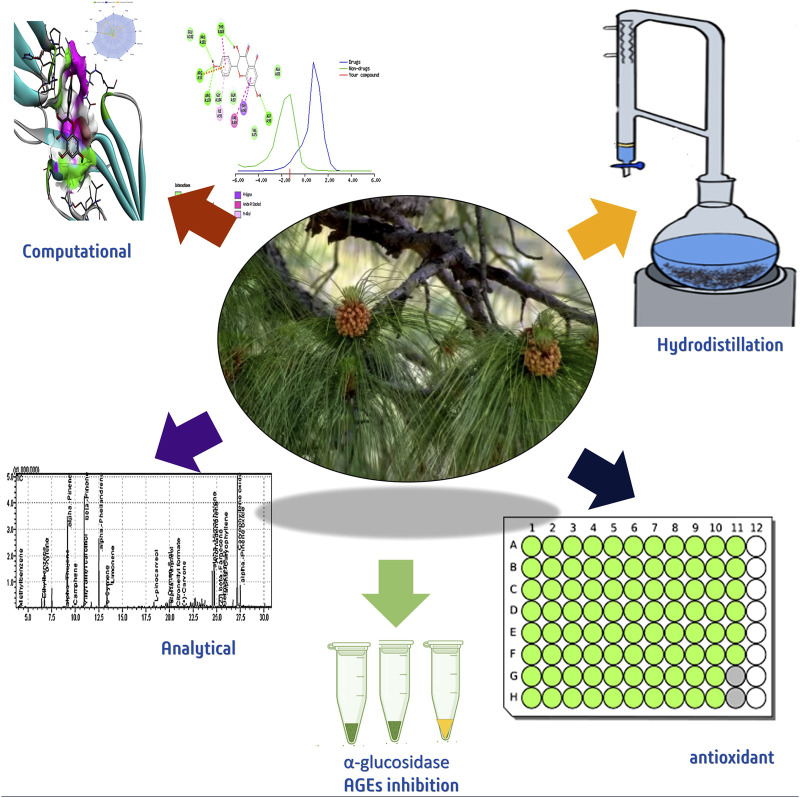

## 1 Introduction

Essential oils (EOs) are mainly derived from aromatic edible plants and contain a variety of plant secondary metabolites (Maffei et al., 2011). The EOs are stored in secretory ducts, oil cells, or cavities within leaves, stems, flowers, and root parts of aromatic plants (Kiran et al., 2013). The EOs may be found bound to carbohydrates as glycosides, and hydrolysis of the glyosidic bonds is required to liberate them from their major source (Ailli et al., 2023). The EOs comprise terpene hydrocarbons, including mono-, di-, and sesquiterpenes; oxygenated derivatives (aldehydes, ketones, epoxides, alcohols, and esters); and sulfur or nitrogen derivatives (Rehman et al., 2007). These complex mixtures of diverse chemical nature pose several bioactive components that encompass a range of biological activities (Ruxton, 2016). Several investigations have confirmed antibacterial, antioxidant, anti-inflammatory, antibiofilm, antifungal, antidiabetic, and anticancer properties of EOs (Dorman and Deans, 2000; Chaieb et al., 2007; Yap et al., 2013; Saharkhiz et al., 2012; Dagli et al., 2015; You et al., 2022). It has been reported earlier that EOs greatly differ in their compositions, and even the composition of EOs extracted from plants of the same species differs in different geographic locations (Benabdelkader et al., 2011). Due to the small size and high penetration power of secondary metabolites in EOs, researchers are showing keen interest in EOs in the quest for promising drug leads (Herman and Herman, 2015).

*Pinus roxburghii* Sarg. (*Pinus roxburghii*; Pinaceae) is a tall tree (up to 30 m) commonly found in Pakistan (Khyber Pakhtunkhwa, Balochistan, and Punjab provinces) that covers a large area of land (Khan et al., 2014; Mehmood et al., 2024). The plant is commonly known as “Chir or Chil Pine” (Amjad et al., 2020). *P. roxburghii* has long been used on the Asian subcontinent to treat conditions like bronchitis, rheumatic pain (Rodriguez et al., 2021), diaphoresis, giddiness, ulcers, inflammation (Kaushik et al., 2012), snake bites, dermatological and topical illnesses (Labib et al., 2016), and gastrointestinal and liver disorders (Kaushik et al., 2012). Recently, researchers have reported anticancer, antimicrobial, antioxidant, anti-inflammatory, anticonvulsant, analgesic, antiparasitic, and antidiabetic properties of *P. roxburghii* (Kaushik et al., 2013; Sajid et al., 2018; Zahid et al., 2023). The EO obtained from *P. roxburghii* has been studied earlier; components including α-pinene, β-phellandrene and β-pinene, δ-3-carene, limonene, and linalool acetate, were identified, and it was proven that this EO can inhibit the activation of the inflammatory transcription factor NF-κB and the expression of the NF-κB-regulated gene (Sajid et al., 2018). Investigators have also reported the antidiabetic potential of *P. roxburghii* (Kaushik et al., 2014; Kaushik et al., 2015; Saber et al., 2021).

Given the rising global burden of diabetes and oxidative stress-related disorders (WHO. Global burden of disease collaborative network, 2024; Chandimali et al., 2025), there is growing interest in identifying natural therapeutic agents with multi-targeted effects. Although several studies have investigated the pharmacological properties of EOs from other *Pinus* species, there is a lack of comprehensive research integrating *in silico* and *in vitro* approaches to validate the bioactivity of *P. roxburghii* essential oil (Ayub et al., 2023). This study aims to bridge this gap by systematically evaluating the antioxidant, antidiabetic, and antiglycation potential of *P. roxburghii* EO. By combining computational docking, molecular dynamics simulations, and *in vitro* biochemical assays, we provide a holistic assessment of its bioactivity, which could contribute to the development of plant-based therapeutics for metabolic disorders. Given its significant medicinal importance, we investigated *P. roxburghii* EO for antioxidant, antidiabetic, and anti-AGE (advanced glycation end product) properties using computational and *in vitro* models to explore its possible applications in diabetes and related co-morbidities. In this study, we provide a holistic assessment of its bioactivity, which could contribute to the development of plant-based therapeutics for metabolic disorders.

## 2 Materials and methods

### 2.1 Chemicals and reagents

All chemicals and reagents used were of analytical-grade, including methylglyoxal (MGO), α-glucosidase (*Saccharomyces cerevisiae*), 2,4,6-Tris (2-pyridyl)-s-triazine (TPTZ), 2,4 dinitrophenyl hydrazine (DNPH), and DPPH (Oxoid, Hampshire, United Kingdom, and Merck, Dorset, United Kingdom). Similarly, sodium azide, D-glucose, and H_2_O_2_ were obtained from Daejung (Siheung-si, Korea). The Folin–Ciocalteu’s reagent was purchased from Sigma (Sigma Aldrich, St. Louis, MI, United States).

### 2.2 Plant collection and authentication and processing

Fresh *P. roxburghii* leaves were collected from the Takht-e-Suleiman range (31°40′57.66″N 69°56′11.64″E; Balochistan province), and herbarium sheets were prepared. These were further authenticated at the Islamabad herbarium, where the specimen was deposited, and an accession ID was obtained (ISL 36502). The plant material was ground and macerated overnight in water in a flask. Afterward, the EO was extracted through hydrodistillation using a Clevenger apparatus. Sodium sulfide was passed through the collected oil to remove traces of water and stored at 4°C until further use.

### 2.3 GC-MS analysis

EO component analysis was performed by using GC-MS (Shimadzu GC 2010; Japan) with a pre-installed autosampler (AOC-20i) and a capillary column (dimensions, 30 m × 0.25 mm id, 0.25-μm film thickness, a DB-5 MS column). The DB-5 MS capillary column was chosen for its high thermal stability, non-polar nature, and ability to efficiently separate volatile and semi-volatile compounds, particularly terpenoids and oxygenated components in *P. roxburghii* essential oil. The temperature of the system oven was initially adjusted to range from 45°C to 90°C, increasing at a rate of 2°C (per minute) and then increasing at a rate of 3°C (per minute) from 91°C to 240°C. After reaching 240°C, temperature was maintained for 5 min. The temperatures of the injector (240°C) and detector (280°C) were maintained at predetermined levels. The temperature program was optimized with a gradual increase (45–240°C) to allow efficient separation of highly volatile monoterpenes at lower temperatures, mid-range oxygenated terpenes at moderate temperatures, and heavier semi-volatiles at the final hold (240°C for 5 min). This process was followed to ensure the accurate identification and resolution of bioactive compounds. An aliquot of EO (0.5 μL) was injected, and helium (1 mL/min) was utilized as a carrier gas to load the sample. The GCMS-QP 2010 Plus (Shimadzu, Japan) instrument was used to analyze and identify GC-MS components. The device operated in electron ionization (EI) mode at 70 eV. Mass units ranging from 35 to 500 AMU were observed. Compounds such as limonene, carvacrol, thymol, α-pinene, β-pinene, β-myrcene, and sabinene were used as a compound mix for identification along with the NIST mass spectrum library (Aziz et al., 2024).

### 2.4 Molecular docking

Potential interactions of ligands with the amino acid residues in the target protein’s active pocket were observed through molecular docking investigations. CAStp (version 3.0) tool was used for the determination of active sites. The 3D structure of compounds was retrieved from the PubChem database, while the target structures such as the receptor for advanced glycation end products (RAGE) (Devi et al., 2016), 3TOP (the C-terminal subunit of human maltase-glucoamylase (MGAM) in complex with the inhibitor acarbose) (Rafey et al., 2022), and 4F5S (the crystal structure of bovine serum albumin) (Soliman et al., 2022) were downloaded from the Protein Data Bank (PDB). Stepwise procedures were used to prepare the target proteins for docking studies, including the addition of charges, extraction of co-crystallized ligands, and the addition of water. Molecular docking was performed using the Lamarckian approach with AutoDock Vina (version 1.2.x (2021–present) (Trott and Olson, 2010). Discovery Studio (version 2019), LigPlot ^+^(version 2.2), and PyMOL (version 2.3.4) were then used to further analyze each pose for interaction analysis. All generated poses were evaluated based on free energy and RMSD values using the following formula:

$$RMSD=\sqrt{\frac{1}{N}\sum_{i=1}^{N}{\left(x_{i}-x_{i,ref}\right)}^{2}+{\left(y_{i}-y_{i,ref}\right)}^{2}+{\left(z_{i}-z_{i,ref}\right)}^{2},}$$

where N is the number of atoms in the ligand; x_i_, y_i_, and z_i_ are the coordinates of atom i in the docked pose; and x_i,ref_, y_i,ref_, and z_i,ref_ are the coordinates of atom i in the reference pose (e.g., the lowest-energy conformation or crystallographic pose).

The RMSD values less than ≤ 2.0 Å indicated a stable binding pose (Fusani et al., 2020).

### 2.5 Total phenolic contents

The Folin–Ciocalteu method was used to determine the total phenolic contents (TPCs) (Chlopicka et al., 2012). Precisely, 0.3 mL of the sample (varying concentrations) was placed in an aliquot, allowed to react with 2.25 mL of Folin’s reagent, and then left undisturbed for 15 min. Subsequently, 2.25 mL of Na_2_CO_3_ (6%) was added and incubated for a further half-hour. Using a UV spectrophotometer, absorbance was determined at 725 nm. The results were expressed as gallic acid equivalent mg (GAE/g) of extract using a gallic acid standard curve with a range of 0–200 μg/mL for standardization.

### 2.6 Antioxidant activities

#### 2.6.1 DPPH assay

Standard procedure was employed to determine the antioxidant activity of the samples (Chlopicka et al., 2012). In brief, 0.5 mL of newly prepared DPPH solution in methanol (0.1 mM) was mixed with the test sample (0.5 mL, different concentrations) and incubated for 30 min (in a dark area to prevent the influence of the influence). Subsequently, absorbance of the reaction mixture was recorded at 517 nm using a UV spectrophotometer (Shimadzu UV-1700; Japan). The control group contained DPPH solution in ethanol with same concentrations without the test sample. Ascorbic acid was used as the standard, and the results were presented as follows:

The outcomes were expressed as

$$\%\text{ Inhibition}=\left(1-\;A_{a}\;/\text{ Ab}\right)\times 100,$$

where A_a_ is the absorbance of the sample, Ab is the absorbance of the control, and ascorbic acid was used as the standard drug.

#### 2.6.2 Hydrogen peroxide assay

The measurement of hydrogen peroxide (H_2_O_2_)-based antioxidant activity was carried out using a modified method (Hussen and Endalew, 2023). In brief, 400 µL of the test sample and 600 µL of the H_2_O_2_ stock solution (2 mM) were combined, and after 10 min, the reaction solution was vortexed, followed by the measurement of absorbance at 230 nm using a UV spectrophotometer (Shimadzu UV-1700; Japan). The control group contained H_2_O_2_ solution in ethanol with the same concentrations without the test sample. The results were expressed using the following equation, where ascorbic acid is considered the reference standard.

$$\%\text{ Inhibition}=\left(1-\;A_{a}\;/\text{ Ab}\right)\times 100,$$

where A_a_ is the absorbance of the sample, Ab is the absorbance of the control, and ascorbic acid was used as the standard drug.

#### 2.6.3 FRAP assay

The ferrous ion reduction power was determined using a modified approach (Song et al., 2023). In brief, fresh FRAP reagent was prepared by combining ferric chloride (20 mM) and TPTZ (10 mM) in a defined ratio (10:1:1) with acetate buffer (300 mM). After reacting with 3 mL of the FRAP reagent, the tested sample (100 µL) was left in a dark area for 30 min. The UV–Vis spectrophotometer (Shimadzu UV-1700; Japan) was then used to record absorbance at 593 nm. The µg equivalents were used to express the results of the FeSO_4_ standard curve, which was further used to determine the ferrous reduction capacity, serving as the control.

### 2.7 Antidiabetic assays

#### 2.7.1 *α-*glucosidase inhibition assay

A modified α-glucosidase inhibition assay was used to record the antidiabetic potential of the test samples (Fatmawati et al., 2023). In brief, the test sample (4 mg–0.039 mg/mL; 50 µL) was mixed with α-glucosidase (*Saccharomyces cerevisiae*) (0.2 U/mL in 0.1 M phosphate buffer at pH 6.8) and incubated for 10 min at 37°C. Subsequently, 100 µL of *p*-nitrophenyl-α-D-glucopyranoside (0.29 mM) was added to the reaction mixture that had already been incubated, and it was kept at 37°C for 30 min. After adding Na_2_CO_3_ (200 mM; 50 µL) to terminate the reaction, the optical density (at 400 nm) was measured. The reaction mixture without the test sample, operating at similar conditions, served as the control. Acarbose served as the reference standard, and % inhibition was determined as follows:

$$\%\text{ Inhibition}=\left(1-\;A_{a}\;/\text{ Ab}\right)\times 100,$$

where A_a_ is the absorbance of the sample, Ab is the absorbance of the control, and acarbose was used as the standard drug. For α-glucosidase, the inhibition criteria were defined as follows: strong inhibition, IC_50_ ≤ 0.02 mg/mL; moderate inhibition, IC_50_ 0.02–0.5 mg/mL; and weak inhibition, IC_50_ > 0.5 mg/mL (criteria based on comparison with standard drug acarbose).

### 2.8 Advance glycation end product assay

#### 2.8.1 BSA-glucose assay

A modified protocol was used to perform AGE assay (non-oxidative mode) (Anwar et al., 2023). In brief, 50 mM of sodium azide (0.02%) was already present in the phosphate buffer (pH 7.4); then, 135 µL of D-glucose (500 mM), 135 µL of BSA (10 mg/mL) and 30 µL of test samples (varying concentrations) were added to the mixture. The mixture was then incubated for a week at 37°C. Subsequently, unreactive contaminants were eliminated, and the additional reaction was terminated by adding trichloroacetic acid (TCA) to the reaction mixture. The spectrofluorometer (FLUOstar Omega^®^, BMG Lab Tech, Aylesbury, United Kingdom) was used to measure the fluorescence intensity (Emission: 370:440 nm and 335:385 nm, respectively). The reaction mixture without the test sample, operating at similar conditions, served as the control. Aminoguanidine was used as the standard in the study, and the following formula was used to calculate the percentage of inhibition:

$$\%\text{ inhibition}=\{1-(\left.\text{Fa }/\;\left(\text{Fb}\right)\right\}\times \;100,$$

where Fa is the florescence of the test sample, Fb is the fluorescence of the control, and IC_50_ values were calculated using a linear plot. Rutin was used as the standard drug. For AGE experiments, the inhibition criteria were defined as follows: significant inhibition, IC_50_ ≤ standard inhibitor; moderate inhibition, IC_50_ between 1 and 2 times the IC_50_ value of the standard; and weak inhibition, IC_50_ > 2 times the IC_50_ of rutin.

#### 2.8.2 BSA-MGO (methylglyoxal) assay

A modified protocol was used to perform AGE assay (oxidative mode) (Anwar et al., 2023). In brief, methylglyoxal (135 μL, 5.75 mM) in the phosphate buffer solution (pH 7.4, 50 mM) with sodium azide (0.02%) was mixed with BSA (135 μL, 10 mg/mL). This solution was combined with 30 μL of test extracts, which were then kept at 37°C for a week. A spectrofluorometer (FLUOstar Omega^®^, BMG Lab Tech, Aylesbury, United Kingdom) was used to measure the fluorescence intensity (excitation: emission at 370:440 nm and 335:385 nm). The reaction mixture without the test sample, operating at similar conditions, served as the control. Aminoguanidine was used as the standard, and the percentage of inhibition was calculated as follows:

$$\%\text{ inhibition}=\{1-(\left.\text{Fa }/\;\left(\text{Fb}\right)\right\}\times \;100,$$

where Fa is the florescence of the test sample, Fb is the fluorescence of the control, and IC_50_ values were calculated using a linear plot. Rutin was used as the standard drug.

#### 2.8.3 β-amyloid protection

For the determination of *β-amyloid* protection, a Congo red solution (0.139 mg/2 mL) was made for this experiment, and 25 µL of it was mixed with 25 µL of the glycated sample (BSA + Glucose). The mixture was then incubated at 30°C for 20 min. Following incubation, 50 µL of distilled water was added to the mixture, and absorbance at the 530 nm wavelength was measured using a spectrophotometer. By comparing the test findings with the control, the tested samples’ protective role was ultimately determined. For interpretation, if the absorbance of the glycated sample is less than that of the control (BSA + glucose), it is reported as protective (Miroliaei et al., 2017).

#### 2.8.4 Free carbonyl group estimation

The presence of a carbonyl group in glycated samples was detected using modified methods, as described earlier (Anwar et al., 2023). A solution of DNPH (2,4 dinitrophenyl hydrazine) with a concentration of 10 mM (mM) was produced using 2.5 M (M) hydrochloric acid (HCl). The glycated sample (500 µL) was subjected to incubation with a solution of DNPH (500 µL) for a duration of 1 h at room temperature. This was then followed by precipitation using 1.0 mL of the TCA solution with a concentration of 20%. The solid was rinsed with a mixture of ethanol and ethyl acetate in equal volumes (1:1 v/v, 1 mL), and the resulting solid was dissolved in urea (1 mL, 6 M). The absorbance of the solution was measured at a wavelength of 365 nm. The concentration of protein carbonyl groups was determined using the molar extinction coefficient (ɛ at 365 nm = 21 mM^-1^cm^−1^) and reported as nM/mg of protein.

### 2.9 Statistical analysis

The biological activity studies were conducted in three independent trials, and the result was expressed as ±SD. A one-way analysis of variance (ANOVA) was conducted, followed by a post hoc Tukey test, with a significance level (*p* value) of less than 0.05.

## 3 Results

### 3.1 GC-MS analysis

**FIGURE 1:**
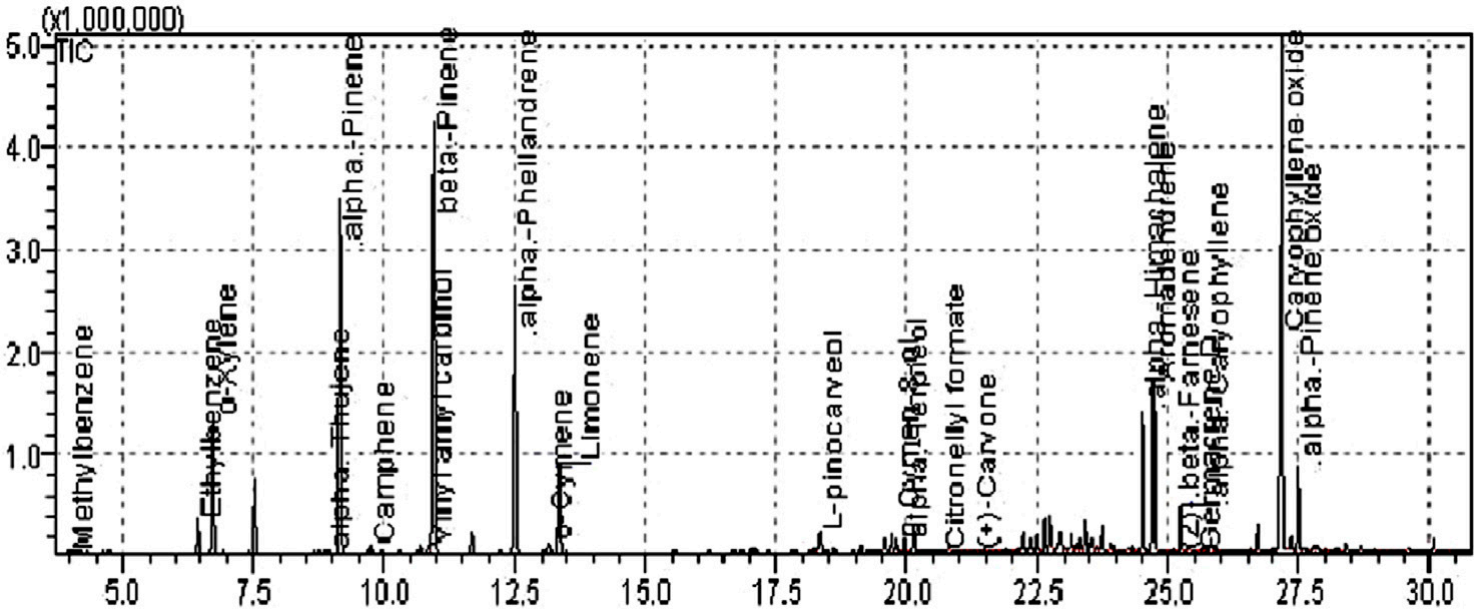
GC-MS chromatogram of the *Pinus roxburghii* essential oil.

**FIGURE 2:**
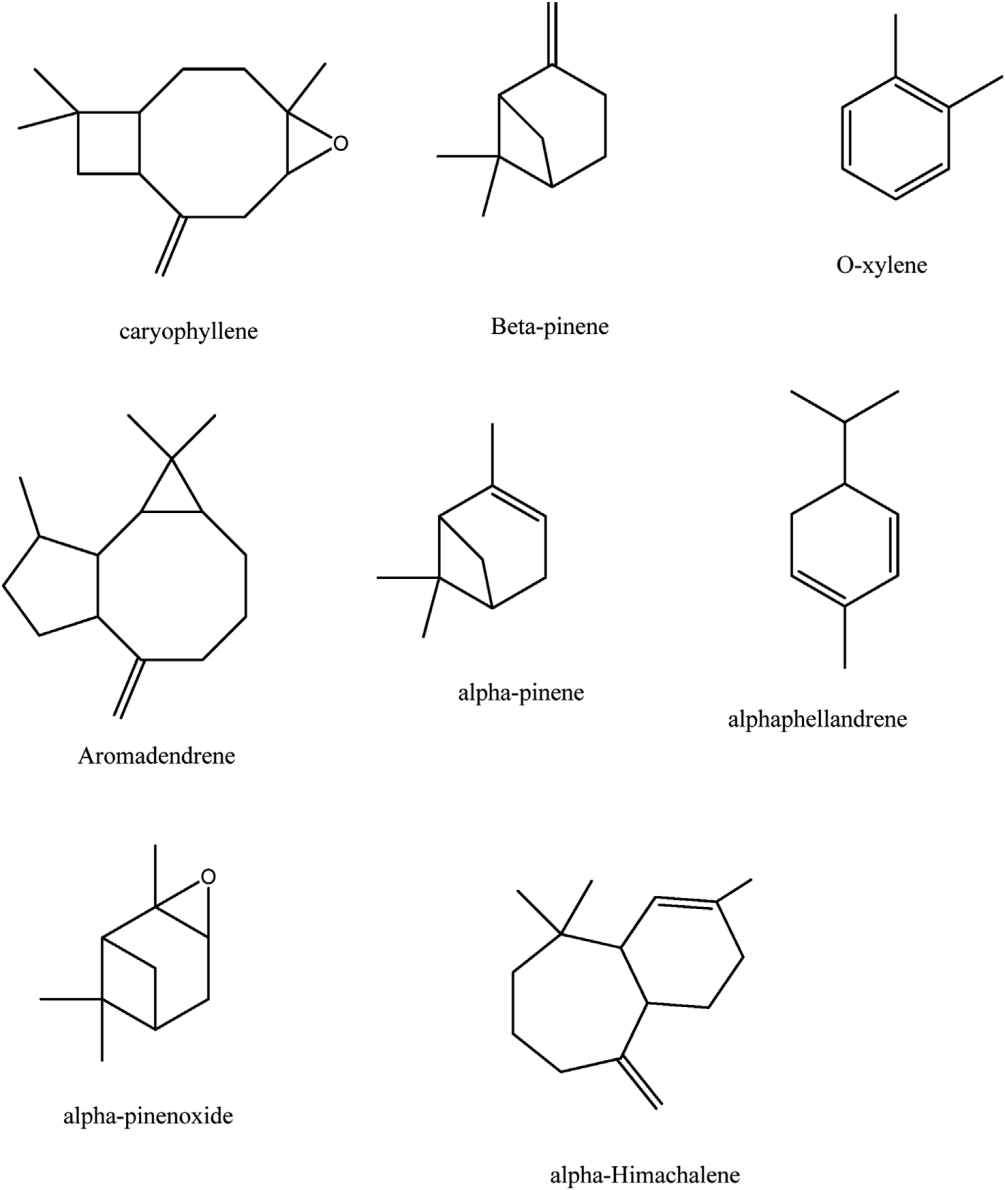
Major chemical constituents of the essential oil of *Pinus roxburghii.*

The GC-MS spectra of *P. roxburghii* were quite complex, and a total of 24 compounds were identified (Table 2). Results revealed the presence of a significant amount of β-pinene (30.08%), followed by caryophyllene oxide (29.37%), O-xylene (8.98%), and aromadendrene (8.29%) (Figure 1). Other compounds such as methylbenzene, ethylbenzene, α-thujone, camphene, (+)-sabinene, amyl vinyl carbinol, α-phellandrene, o-cymene, limonene, L-pinocarveol, p-cymen-8-ol, α-terpineol, citronellyl formate, (+)-carvone, α-himachalene, and β-farnesene (Tables 1, 2; Figure 2).

**TABLE 1:** GC-MS profile of the *Pinus roxburghii* essential oil.

S.No	ID No.	Name	R. time	Area	Concentration %
1	1	Methyl benzene	3.963	6,136	0.01
2	2	Ethylbenzene	6.449	836,320	1.99
3	3	o-Xylene	6.711	3,772,219	8.98
4	4	α-thujene	8.906	11,387	0.03
5	5	α-pinene	9.154	1,988,904	4.73
6	6	Camphene	9.742	28,663	0.07
7	7	(+)-Sabinene	10.846	45,489	0.11
8	8	β-pinene	10.948	12,638,607	30.08
9	9	Amyl vinyl carbinol	10.846	45,489	0.11
10	14	α-phellandrene	12.490	1,416,686	3.37
11	16	o-cymene	13.139	96,804	0.23
12	17	Limonene	13.353	409,220	0.97
13	25	L-pinocarveol	18.326	61,686	0.15
14	29	p-cymen-8-ol	19.790	109,268	0.26
15	30	α-terpineol	19.968	33,922	0.08
16	31	Citronellyl formate	20.695	2,553	0.01
17	34	(+)-Carvone	21.285	5,231	0.01
18	45	α-himachalene	24.516	2,865,275	6.82
19	46	Aromadendrene	24.721	3,480,718	8.29
20	50	β-farnesene	25.153	3,940	0.01
21	51	α -caryophyllene	25.256	140,687	0.33
22	53	Germacrene D	25.561	1821	0.00
23	59	Caryophyllene oxide	27.152	12,338,343	29.37
24	62	α-pinene oxide	27.494	1,672,452	3.98

**TABLE 2:** Drug-likeness scores and SMILES of major compounds from the *P. roxburghii* essential oil.

S.No	Compound	Score	SMILES
1	β-pinene	−1.39	CC1(C2CCC(=C)C1C2)C
2	Caryophyllene oxide	−1.50	CC1(CC2C1CCC3(C(O3)CCC2 = C)C)C
3	Aromadendrene	−1.30	CC1CCC2C1C3C(C3(C)C)CCC2 = C
4	o-xylene	−1.60	CC1 = CC = CC = C1C
5	α-phellandrene	−1.23	CC1 = CCC(C=C1)C(C)C
6	α -himachalene	−1.24	CC1 = CC2C(CC1)C (=C)CCCC2(C)C

### 3.2 Drug-likeness ADMET analysis

**FIGURE 3:**
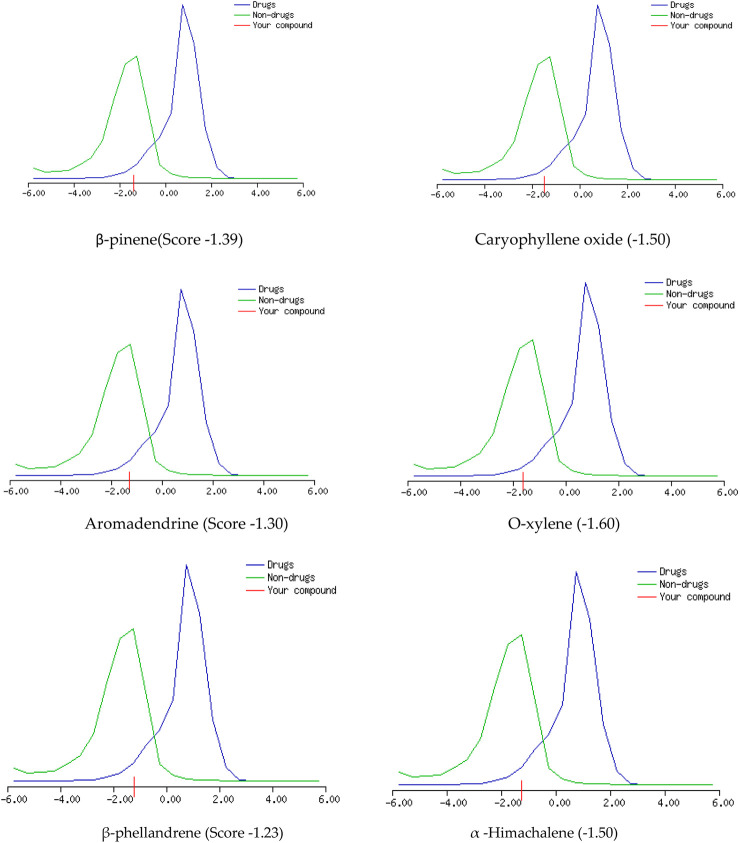
Drug-likeness scores of major components from *P. roxburghii*.

**FIGURE 4:**
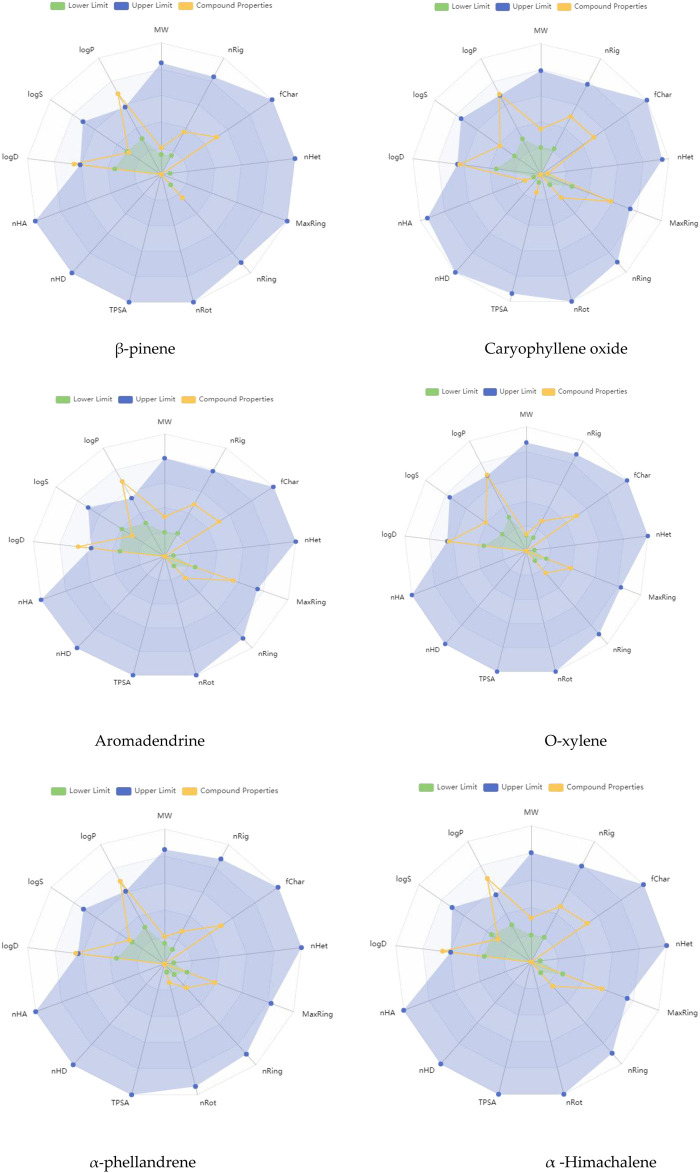
ADMET properties in radar view covering major components of *P. roxburghii*.

The GC-MS spectra of *P. roxburghii* EO showed a large concentration of β-pinene, caryophyllene oxide, aromadendrene, o-xylene, α-phellandrene, and α-himachalene. We, therefore, decided to analyze these compounds for *in silico* characterization. The drug-likeness score of all components showed deviation from the set value (−1 to +1); however, values were only a slight different from the range in the case of α-phellandrene (−1.23) and α-himachalene (−1.24). Lipinski’s rule was followed by all compounds except aromadendrene, which showed a violation in the case of Log p (5.22) (Lipinski et al., 1997); however, still, it was only a minor deviation (Tables 1, 2; Figure 3, 4). The ADMET analysis of *P. roxburghii* indicated that all tested compounds showed low absorption from the oral route except caryophyllene oxide (high) and that all tested compounds were able to cross blood–brain barrier (BBB) except α-himachalene (Table 4). None of the molecules affected P-gp substrate and were mostly metabolized in the liver. Detailed ADMET results are mentioned in Table 4.

**TABLE 3:** Lipinski properties of compounds from the *P. roxburghii esse*ntial oil.

S.No	Compound	Molecular weight <500 Da	Log p < 5	H bond Donor (5)	H-bond acceptor <10	No. of violations
1	β-pinene	136.13	4.14	0	0	0
2	Caryophyllene oxide	220.18	3.97	0	1	0
3	Aromadendrene	204.19	5.22	0	0	1
4	o-xylene	106.08	3.00	0	0	0
5	α-phellandrene	136.13	4.01	0	0	0
6	α -himachalene	204.19	5.30	0	0	0

**TABLE 4:** Pharmacokinetic parameter of major components from the *P. roxburghii* essential oil.

Parameter	β-pinene	Caryophyllene oxide	Aromadendrene	o-xylene	α-phellandrene	α -himachalene
GIT absorption	Low	High	Low	Low	Low	Low
BBB permeant	Yes	Yes	Yes	Yes	Yes	No
P-gp substrate	No	No	No	No	No	No
CYP1A2 inhibitor	No	No	Yes	No	No	No
CYP2C19 inhibitor	No	Yes	yes	No	No	Yes
CYP2C9 inhibitor	Yes	yes	Yes	No	No	Yes
CYP2D6 inhibitor	No	No	Yes	No	No	No
CYP3A4 inhibitor	No	No	No	No	No	No
Log *K*_p_ (skin permeation)	−4.18 cm/s	−5.12 cm/s	−4.20 cm/s	−4.73 cm/s	−4.85 cm/s	−4.38 cm/s

### 3.3 Molecular docking

**FIGURE 5:**
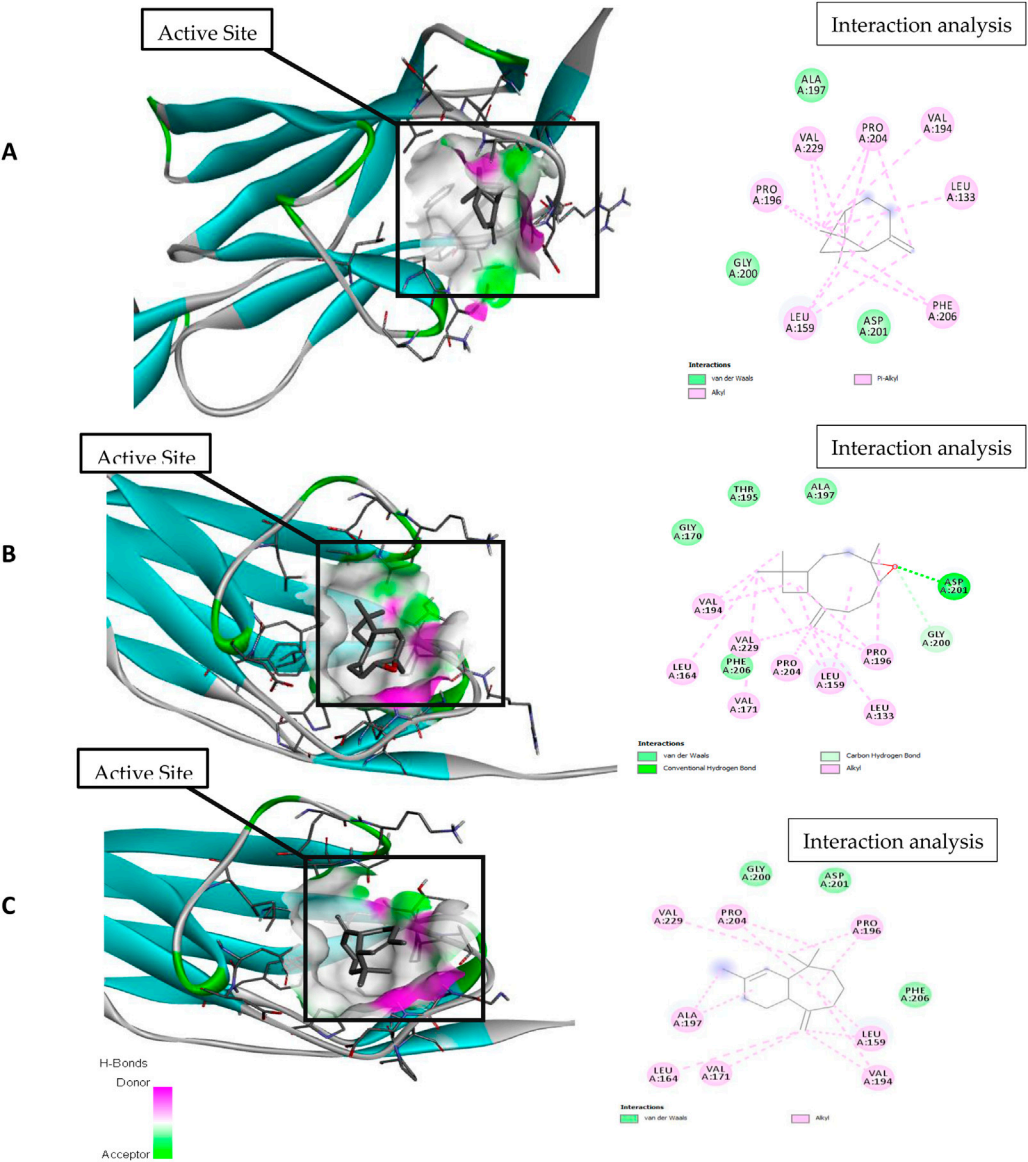
Molecular docking and interaction analysis of β-pinene **(A)**, caryophyllene oxide **(B)**, and α-himachalene **(C)** with 3CJJ from the *P. roxburghii* essential oil.

**FIGURE 6:**
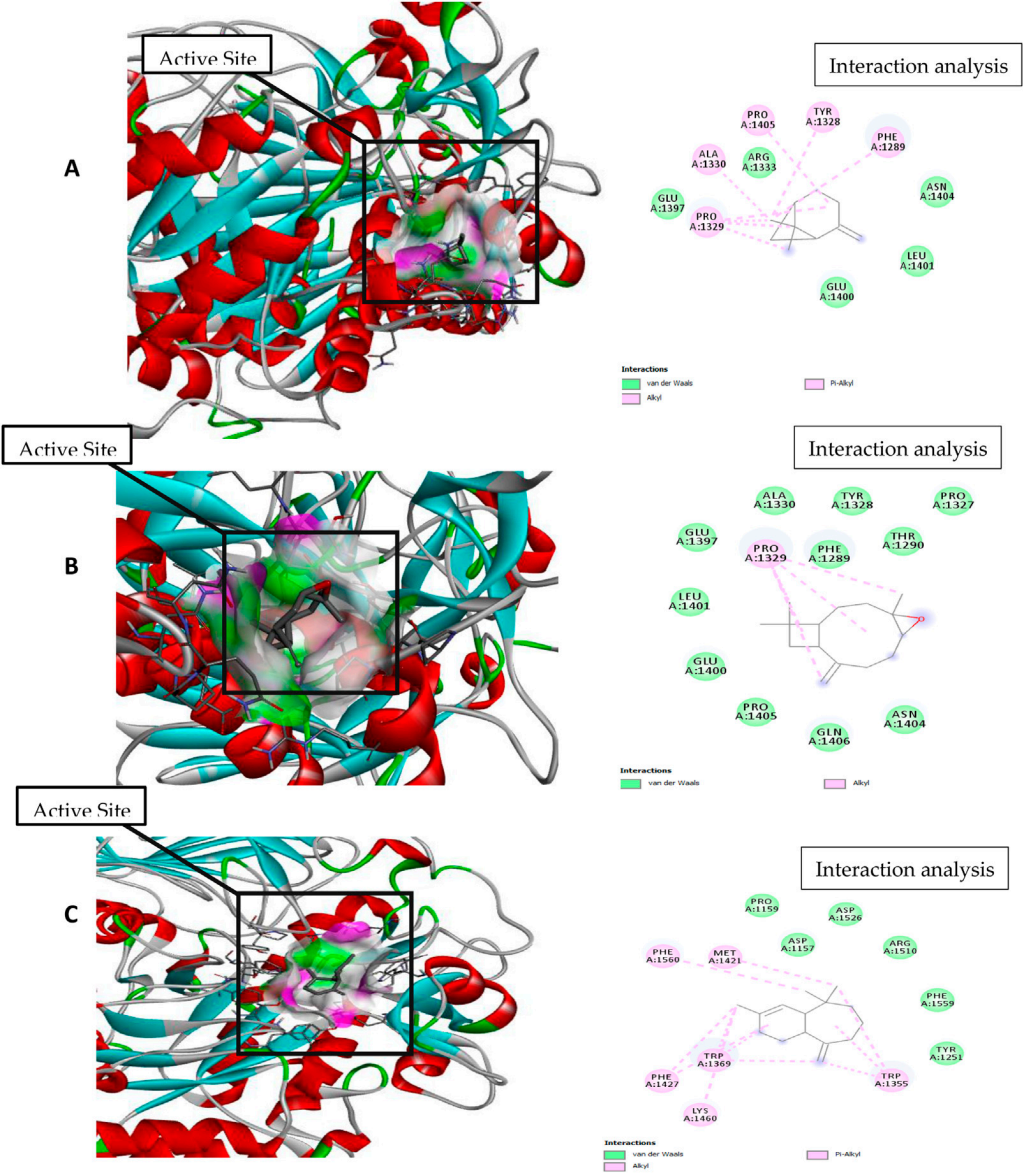
Molecular docking and interaction analysis of β-pinene **(A)**, caryophyllene oxide **(B)**, and α-himachalene **(C)** with 3TOP from the *P. roxburghii* essential oil.

**FIGURE 7:**
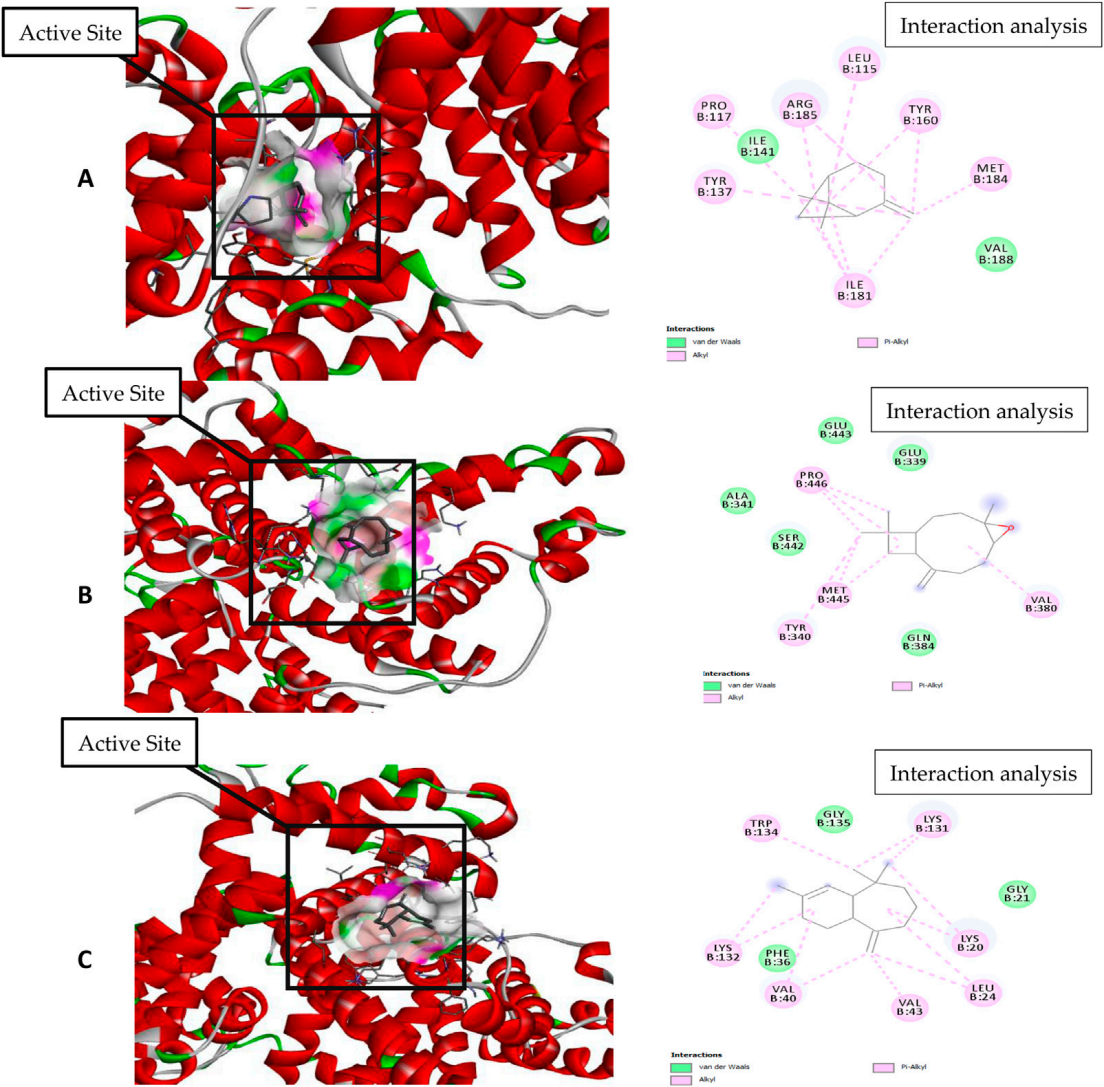
Molecular docking and interaction analysis of β-pinene **(A)**, caryophyllene oxide **(B)**, and α-himachalene **(C)** with 4F5S from the *P. roxburghii* essential oil.

For molecular docking, target proteins 3CJJ, 3TOP, and 3F5S were selected for ligand docking. Based on the relative abundance of components in *P. roxburghii* EO, β-pinene, caryophyllene oxide, and α-himachalene were selected for molecular docking investigations. Based on the findings, it was evident that the docking complex in 3CJJ and caryophyllene oxide was stabilized by both H-bonding and hydrophobic interactions, whereas in all other tested molecules, hydrophobic forces were mainly observed (Table 5; Figure 5). During docking with 3TOP and 4F5S receptors, hydrophobic forces played a dominant role in protein folding (Table 5; Figures 6, 7).

**TABLE 5:** Docking score and interaction analysis of major components in the *P. roxburghii* essential oil.

Ligand	Binding free energy ΔG (kJ mol^‒1^)	Pose rank	No of H bonds	H bond interaction residues	Other interaction residues
3CJJ					
β-pinene	−5.4	1	0	0	Leu 159, Gly200, Pro196, Val229, Pro204, Ala197, Val194, Leu133, Phe206, Asp201
Caryophyllene oxide	−7.5	1	1	Asp201	Pro204, Val171, Phe206, Val229, Leu164, Val194, Gly170, Thr195, Ala197, Gly200, Pro196, Leu133, Leu159
α-himachalene	−6.5	1	1	0	Val171, Leu164, Ala197, Val229, Pro204, Gly200, Asp 201, Pro296, Phe206, Leu159, Val194
3TOP					
β-pinene	−5.9	1	0	0	Glu1400, Pro1329, Glu1397, Ala1330, Arg1333, Pro1405, Tyr1328, Phe1289, Asn1404, Leu1401
Caryophyllene oxide	−6.1	1	0	0	Gln 1406, Pro1405, Glu1400, Leu1401, Glu1397, Pro1329, Ala1330, Phe1289, Tyr1328, Thr1290, Pro1327, An1404
α-himachalene	−6.8	1	0	0	Lys1460, Phe1427, Trp1369, Phe1560, Met1421, Asp1157, Pro1159, Asp1526, Arg1510, Phe1559, Tyr1251, Trp1355
4F5S					
β-pinene	−5.9	1	0	0	Ile181, Tyr137, Ile141, Pro117, Leu115, Arg185, Leu115, Tyr160, Me184, Val188
Caryophyllene oxide	−5.9	1	0	0	Gln384, Tyr340, Met445, Ser442, Ala341, Pro446, Glu443, Glu339, Val380
α-himachalene	−7.7	1	0	0	Val40, Phe36, Lys132, Trp134, Gly135, Lys131, Gly21, Lys20, Leu24, Val43

### 3.4 Biological evaluation

**FIGURE 8:**
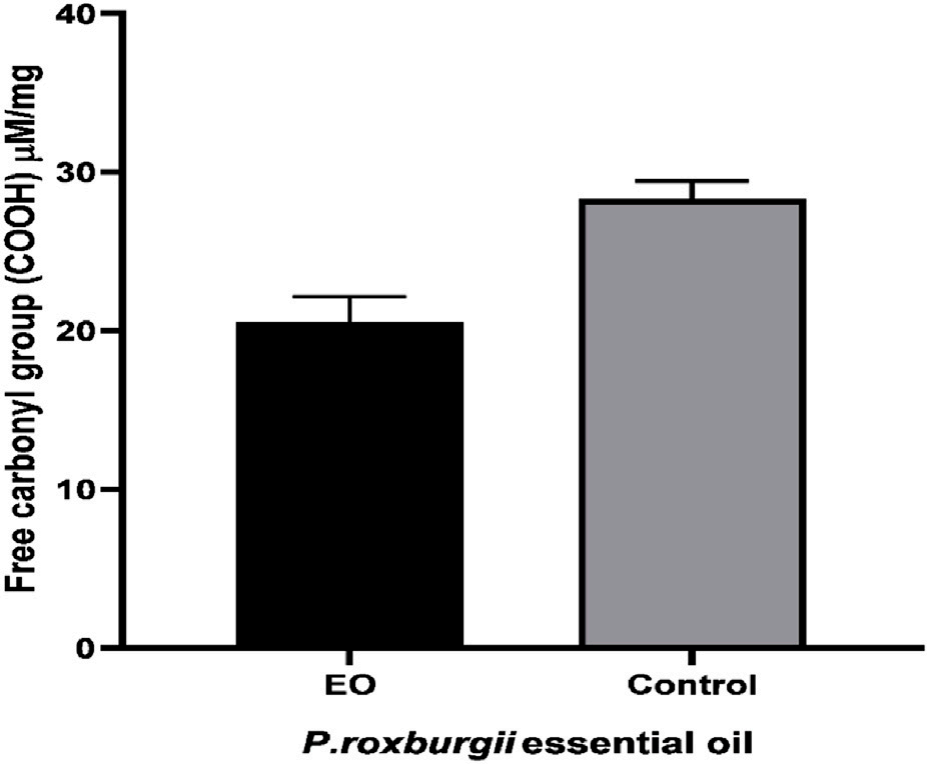
Estimation of free carbonyl entrapment by the *P. roxburghii* essential oil.

The *P. roxburghii* EO was assayed for the presence of phenolic compounds using the Folin–Ciocalteu reagent*.* The gallic acid equivalents *(*mg/g GAE) were calculated using the gallic acid calibration curve. It was noticed that *P. roxburghii* (0.263 ± 0.45 mg/g GAE) possessed slight phenolic contents (Table 6). Further significantly high antioxidant potential in all assays, including FRAP (312 ± 14.6 µg), DPPH (IC_50_ 14.2 ± 0.62 μg/mL), and H_2_O_2_ (73.3 ± 1.7 inhibition) assays, was recorded (Table 6). The *P. roxburghii* EO inhibition of α-glucosidase (IC_50_ 0.12 mg/mL) was considered moderate after comparison with the standard drug acarbose (IC_50_ 0.0014 mg/mL), which is eight times less potent (Table 7). In AGE assays, *P. roxburghii* EO showed inhibition in both non-oxidative (BSA-glucose; IC_50_ 0.052 mg/mL) and oxidative modes (BSA-MGO; IC_50_ 1.61 mg/mL). These findings were considered significant (BSA Glucose) and moderate (BSA-MGO) based on the criteria set compared with the standard used (rutin) (Table 7). Furthermore, during mechanistic studies, the EOs were analyzed for their potential role in the protection of β-amyloid sheet formation and fructosamine entrapment. The *P. roxburghii* EOs were able to possess a moderate protective effect against β-amyloid formation (Table 8). The DTNB molar extinction coefficient value (ɛ at 365 nm = 21 mM^−1^ cm^−1^) was used for free carbonyl entrapment (µM), and it was observed that the *P. roxburghii* EO *S.* presented moderate entrapment of carbonyls (Figure 8).

**TABLE 6:** Phenolic contents and antioxidant activity of the tested *P. roxburghii* essential oil.

Sample name	Total phenolics (mg GAE/g)	H_2_O_2_ % inhibition^a^	FRAP value µg	DPPH IC_50_ µg/mL
*P. roxburghii*	0.263 ± 0.45	73.3 ± 1.7	312 ± 14.6	14.2 ± 0.62
Ascorbic acid	—	73.2 ± 0.4	266.1 ± 1.8	20.2 ± 1.2

^a^ 120 μg/mL.

**TABLE 7:** Advance glycation end products and α-glucosidase inhibitory potential of the *P. roxburghii* essential oil.

Sample	BSA-Glc (IC_50_ mg/mL)	BSA-MGO (IC_50_ mg/mL)	α-glucosidase (IC_50_ mg/mL)
*P. roxburghii*	0.052 ± 0.0025	1.61 ± 0.0013	0.12 ± 0.0023
Standard	0.10^1^ ± 0.0031	0.82^1^ ± 0.0024	0.014^2^ ± 0.0027

^1^rutin ^2^acarbose, Fract = fraction. For α-glucosidase, strong inhibition: IC_50_ ≤ 0.02 mg/mL; moderate inhibition: IC_50_ 0.02–0.5 mg/mL; and weak inhibition: IC_50_ > 0.5 mg/mL (criteria based on comparison with the standard drug acarbose). For AGE experiment criteria, significant inhibition: IC_50_ ≤ standard inhibitor; moderate inhibition: IC_50_ between 1 and 2 times the IC_50_ value of the standard; and weak inhibition: IC_50_ > 2 times the IC_50_ value of rutin.

**TABLE 8:** Detection of the protective role of the *P. roxburghii* essential oil against β-amyloid formation.

Essential oil	Absorbance	Remark*
*P. roxburghii*	0.34	Moderate
Control	0.10	

^*^ The test sample is considered good if it has protective effect on β-sheets of the protein (BSA). Thus, the results must be less that control, i.e., BSA + Glucose. Protective means very good antiglycation properties and Moderate means moderate activity (Final tested concentration = 0.8 mg/mL).

## 4 Discussions

The GC-MS analysis of *P. roxburghii* EO confirmed the presence of cyclic monoterpenes, including β-pinene (abundant terpenoids). Earlier investigations have suggested a bit of diversity in components and their concentrations (Satyal et al., 2013; Labib et al., 2017; Ayub et al., 2025). The essential oil composition varies in general due to certain environmental factors (such as climate, soil, and altitude), plant development stages, and extraction tissue (Eaissi et al., 2011; Benomari et al., 2023). Furthermore, harvesting time and post-harvest processing can also alter the concentration of volatile components through oxidative or enzymatic activities (Sun et al., 2022).

The most abundant component identified from GC-MS analysis was further used in computational assays including drug-likeness, ADMET, and docking analysis. The drug-likeness software generally uses algorithms that specifically determine whether the chemical entity under question poses physical and chemical properties to be considered a drug molecule (Bickerton et al., 2012) Based on Lipinki’s rule and drug-likeness scores (molinspiration tool), it was evident that aromadendrin showed a slight deviation (+1 to −1), whereas higher drug-likeness scores were recorded with other tested compounds. The ADMET analysis of *P. roxburghii* EO showed interesting results, and all tested compounds showed lower absorbance rates from the oral route except for caryophyllene oxide (high). Likewise, all compounds were able to cross the BBB except α-himachalene (Figure 4). Compounds with BBB permeability can potentially influence central nervous system (CNS) functions, highlighting the importance of evaluating their neurological safety profiles (Velásquez-Jiménez et al., 2021; Isabel et al., 2024). Based on the existing literature, these compounds have not shown significant adverse CNS effects in previous experimental studies; however, further *in vivo* assays are recommended for comprehensive safety evaluation. Similarly, none of the tested molecules were P-gp substrate (responsible for exclusion or transport of drugs), which means that none of these molecules can be actively removed from cells by P-glycoprotein. Metabolism in the liver is mainly regulated by CYP450 enzymes, and drug molecules that serve as substrate for these enzymes will be metabolized in the liver and vice versa (Han et al., 2019). It is obvious from our *in silico* analysis that few of the compounds acted as substrates, and others were inhibitors of these enzymes, which shows that these molecules may not be metabolized in the liver. Log kp is an indicator of skin permeation, and the more negative the log *kp* (with *K*_p_ in cm/s), the lower the skin permeation of the tested molecule (Daina et al., 2017). Based on the findings of our *in silico* analysis, it was evident that all tested molecules had low skin permeation.

In docking studies for 3CJJ, caryophyllene oxide showed the best complex formation with the target protein, exhibiting a high free energy [−7.5 ΔG (kJ mol^‒1^)]. This interaction was stabilized by H-bond formation through Asp201. The protein folding was protected by hydrophobic interactions that occurred through Van der Waal’s π-alkyl and alkyl interactions through Leu 159, Gly200, Pro196, Val229, Pro204, Ala197, Val194, Leu133, Phe206, and Asp201 amino acid residues. α-himachalene was also able to form a strong interaction with 3CJJ with high free energy [−6.5 ΔG (kJ mol^‒1^)]. The complex formation was mainly supported through hydrophobic interactions (Van der Waal’s and alkyl interactions) involving Val171, Leu164, Ala197, Val229, Pro204, Gly200, Asp 201, Pro296, Phe206, Leu159, and Val194 amino acid residues (Table 1; Figure 1). Finally, β-pinene docking with 3CJJ showed a stable complex formation with significantly high free energy [−5.4 ΔG (kJ mol^‒1^)]. In this study, no H-bond formation occurred between the ligand and receptor proteins, and mainly hydrophobic interactions were observed with Leu 159, Gly200, Pro196, Val229, Pro204, Ala197, Val194, Leu133, Phe206, and Asp201.

In docking studies for 3TOP, α-himachalene showed the best docking with the target protein, exhibiting a high free energy [−6.8 ΔG (kJ mol^‒1^)]. In this study, protein folding was protected by hydrophobic interactions that occurred through Van der Waal’s π-alkyl and alkyl interactions, including 12 amino acids, namely, Lys1460, Phe1427, Trp1369, Phe1560, Met1421, Asp1157, Pro1159, Asp1526, Arg1510, Phe1559, Tyr1251, and Trp1355 amino acids. Caryophyllene oxide was also able to show a strong interaction with receptors with high free energy [−6.1 ΔG (kJ mol^‒1^)]. The complex formation was mainly supported through hydrophobic interactions (Van der Waal’s and alkyl interactions) involving 12 amino acids, namely, Gln 1406, Pro 1405, Glu 1400, Leu 1401, Glu 1397, Pro 1329, Ala 1330, Phe 1289, Tyr 1328, Thr 1290, Pro 1327, and An 1404, amino acid residues. Similarly, β-pinene docking with 3TOP showed a stable complex formation with significantly high free energy [−5.9 ΔG (kJ mol^‒1^)]. In this study, no H-bond formation occurred between the ligand and receptor proteins, and mainly hydrophobic attachments were recorded with Glu1400, Pro1329, Glu1397, Ala1330, Arg1333, Pro1405, Tyr1328, Phe1289, Asn1404, and Leu1401. The interaction of the bovine serum albumin receptor 5NN8 with the major components of *P. roxburghii* EO was studied. β-pinene showed the best docking with the target protein with a high free energy [−5.5 ΔG (kJ mol^‒1^)]. Protein folding was protected by hydrophobic interactions that were formed through Van der Waal’s π-alkyl and alkyl interactions, including five amino acids, namely, Glu430, Leu431, Arg385, Met427, and Val388. Similarly, α-himachalene interacted with receptors with a high free energy [−5.4 ΔG (kJ mol^‒1^)]. In this case, neighboring amino acids included Arg168, Leu167, Thr151, Pro186, Ala150, Thr149, and Asn140 (Table 5; Figure 7). β-pinene docking with 5NN8 also showed a stable complex formation with a significantly high free energy [−4.7 ΔG (kJ mol^‒1^)]. The complex is mainly supported by hydrophobic interactions that involve Asp163, Ile164, Arg190, Lys162, Asp243, Asp185, and Asn188 (Table 5; Figure 7).

The *P. roxburghii* EOs were observed as rich in phenolic contents, and this was due to the presence of diverse monoterpenes as we noticed and reported earlier (Labaib et al., 2017; Ayub et al., 2023). High phenolic contents are mainly associated with strong antioxidant potential since these compounds have a strong affinity for free radicals and deactivate them, thereby reducing oxidative stress (Aryal et al., 2019). The EOs were, therefore, assessed for antioxidant potential through different analytical mechanisms, including FRAP, H_2_O_2_, and DPPH assays. It was observed that *P. roxburghii* EO exhibited oxidative stress-lowering capacity. Similar findings were reported in earlier investigations on essential oils in different geographical locations (Djerrad et al., 2015; Mehmood et al., 2024; Ancuceanu et al., 2024). Various pathological conditions trigger excessive production of ROS, which are the main reason for the increase in oxidative stress (Phaniendra et al., 2015). Reports have suggested that elevated oxidative burden unfavorably affects various cellular functions, thereby leading to diseased conditions (García-Sánchez et al., 2020). Similar to the case of diabetes mellitus, high oxidative stress results in impaired β-cell function, leading to disordered glucose regulation in the body (Caturano et al., 2023). Thus, the antioxidative potential of *P. roxburghii* EO can be useful for the management of diabetes due to significant antioxidant effects.

Among various mechanisms of diabetes mellitus, the consideration of the α-glucosidase enzyme in the intestinal lumen is very important. We investigated four EOs for potential α-glucosidase inhibition, and moderate α-glucosidase inhibition was recorded in the case of *P. roxburghii* EO. Earlier investigations have reported a significant α-glucosidase inhibition but slightly lower than that of our investigation, which is mainly attributed to geographical diversities (Maurya et al., 2022) and differences in terpenoid composition. The terpenoid contents mainly contribute toward diverse biological activities, including α-glucosidase (Masyita et al., 2022). Among these, mono-terpenoids are very important since they have very low affinity for water (hydrophobic) and thereby deactivate enzymes, especially α-glucosidase, thus playing a key role in the absorption of carbohydrates from gut mucosa (Kozioł et al., 2014).

Persistent hyperglycemia generally leads to the development of several micro and macrovascular complications, primarily due to the production of advanced glycation end products (AGEs) through non-enzymatic pathways (Lee et al., 2022). Thus, an ideal drug lead should not only reduce the development of hyperglycemia but also slow down AGE formation. We thus investigated EO for its anti-AGE activity and inhibition mechanisms. A significant moderate inhibition of AGEs in both oxidative and non-oxidative modes was observed, and to the best of our knowledge, no previous data are available on the same essential oil. Despite this, several investigations have confirmed AGE inhibitory potential of essential oils rich in monoterpenoids including α-pinene, β-pinene, and caryophyllene oxide (Thomas and Essien, 2020; Yadav et al., 2023; El et al., 2024), and this was mainly correlated with its high terpenoid contents and strong antioxidant potential in scavenging free radicals (Khan et al., 2020; Rhizlan et al., 2024).

The AGE formation may also result in protein dysfunction due to interference with β-sheets of the protein (Lannuzzi et al., 2014). The β-amyloid assay was performed, as described earlier, and the test sample was considered as having a “protective effect” if its absorbance was less than that of the control, i.e., BSA + Glucose. Protective means very good antiglycation properties, and moderate means moderate protection against glycation. As obvious, the EO exhibited lower absorbance compared to the control. Likewise, it showed significant carbonyl entrapment. Thus, a drug candidate with a protective effect against β-amyloid formation and carbonyl entrapment is desirable for maintaining protein function. During persistent hyperglycemia, free carbonyls and their intermediates are generated, which are the main promoters of AGE formation (Schalkwijk et al., 1999).

With these findings, the *P. roxburghii* EO can be further investigated for *in vivo* validation. However, the volatile nature of essential oils and geographical variations can limit its effectiveness. We, therefore, propose formulation design and synergy studies with other compounds, which can be very helpful for the pharmaceutical industry.

## 5 Conclusion

The major compounds of *P. roxburghii* EO were docked against selected transcriptional regulators 3CJJ, 3 TOP, and 4F5S, revealing significant interactions within the active pockets. The findings of this investigation confirmed the significant antioxidant potential of *P. roxburghii* EO, which is rich in terpenoid moieties. The EO significantly contributed to the inhibition of α-glucosidase and AGEs through β-amyloid protective effects and free carbonyl entrapment. It was, therefore, concluded that *P. roxburghii* EO can be effectively employed for the management of diabetes and its associated complications. These findings can be helpful in future formulation design and will provide a potential integrated approach for diabetes and its comorbidities.

## Data Availability

The original contributions presented in the study are included in the article/supplementary material, further inquiries can be directed to the corresponding author.
